# Extinction of Fear Memory Attenuates Conditioned Cardiovascular Fear Reactivity

**DOI:** 10.3389/fnbeh.2018.00276

**Published:** 2018-11-13

**Authors:** Adam P. Swiercz, Antonia V. Seligowski, Jeanie Park, Paul J. Marvar

**Affiliations:** ^1^Department of Pharmacology and Physiology and Institute for Neuroscience, George Washington University, Washington, DC, United States; ^2^McLean Hospital, Harvard Medical School, Belmont, MA, United States; ^3^Atlanta VA Medical Center, Division of Renal Medicine, Emory University School of Medicine, Atlanta, GA, United States; ^4^Department of Psychiatry and Behavioral Sciences, George Washington University, Washington, DC, United States

**Keywords:** PTSD, fear memory, Pavlovian fear conditioning, cardiovascular disease, extinction, physiological hyperarousal

## Abstract

Post-traumatic stress disorder (PTSD) is characterized by a heightened emotional and physiological state and an impaired ability to suppress or extinguish traumatic fear memories. Exaggerated physiological responses may contribute to increased cardiovascular disease (CVD) risk in this population, but whether treatment for PTSD can offset CVD risk remains unknown. To further evaluate physiological correlates of fear learning, we used a novel pre-clinical conditioned cardiovascular testing paradigm and examined the effects of Pavlovian fear conditioning and extinction training on mean arterial pressure (MAP) and heart rate (HR) responses. We hypothesized that a fear conditioned cardiovascular response could be detected in a novel context and attenuated by extinction training. In a novel context, fear conditioned mice exhibited marginal increases in MAP (∼3 mmHg) and decreases in HR (∼20 bpm) during CS presentation. In a home cage context, the CS elicited significant increases in both HR (100 bpm) and MAP (20 mmHg). Following extinction training, the MAP response was suppressed while CS-dependent HR responses were variable. These pre-clinical data suggest that extinction learning attenuates the acute MAP responses to conditioned stimuli over time, and that MAP and HR responses may extinguish at different rates. These results suggest that in mouse models of fear learning, conditioned cardiovascular responses are modified by extinction training. Understanding these processes in pre-clinical disease models and in humans with PTSD may be important for identifying interventions that facilitate fear extinction and attenuate hyper-physiological responses, potentially leading to improvements in the efficacy of exposure therapy and PTSD–CVD comorbidity outcomes.

## Introduction

Post-traumatic stress disorder (PTSD) is a debilitating psychiatric disorder in which over-generalization of fear and impaired fear extinction recall can lead to a permanent state of hyperarousal and emotional numbing that can negatively impact daily life (Desmedt et al., 2015). PTSD is in part characterized by an inability to adequately suppress fear responses under safe conditions (Jovanovic et al., 2010; Sijbrandij et al., 2013) and is often accompanied by exaggerated physiological symptoms [e.g., increased heart rate (HR), blood pressure, and sympathetic drive] (American Psychiatric Association, 2013; Edmondson et al., 2013; Vaccarino et al., 2013; Park et al., 2017). Enhanced acute stress responses may contribute to increased rates of acute cardiac events and cardiovascular disease (CVD) risk in PTSD patients (Edmondson et al., 2013; Edmondson and von Känel, 2017; Myers, 2017), however, the underlying physiological mechanisms remain unclear. Further examination of altered cardiovascular reactivity that occurs in states of fear is therefore required to better understand the link between PTSD and CVD.

Classical rodent and human models of fear conditioning are commonly used to study both the expression and extinction of learned fear, providing practical methods to identify PTSD biomarkers and prevention strategies. For example, extinction training is believed to be analogous to exposure therapy, which is one of the most effective therapeutic treatments for PTSD, phobias, and anxiety disorders (Morrison and Ressler, 2014). Extinction training results in the formation of a new extinction memory, and a gradual reduction of conditioned response (Quirk, 2002). Extinction recall occurs when the extinction memory is retrieved and expressed at a later time (Quirk et al., 2000; Milad et al., 2009). Due to its potential as a therapeutic target, many efforts have recently been made to discover treatments that may strengthen or facilitate extinction recall (Bukalo et al., 2014; Bowers and Ressler, 2015). Critical to these efforts is the ability to accurately measure extinction-specific changes to conditioned responses.

While most assessments of extinction in animal models rely entirely on changes in freezing behavior, conditioned cardiovascular responses have previously been shown to serve as important physiological correlates of fear learning (Gaburro et al., 2011). For example, rodent studies using radiotelemetry demonstrate that HR (Tovote et al., 2005b), HR variability (Stiedl et al., 2009), and blood pressure (Hsu et al., 2012) are reliable indicators of fear memory acquisition that can be used to distinguish between non-specific and associative threat responses. However, few studies have examined the effects of fear extinction on cue-dependent conditioned cardiovascular responses. Within these studies, the focus has primarily been on reductions in HR reactivity (Stiedl, 1999; Stiedl et al., 2009; Camp et al., 2012; Hager et al., 2014).

Under certain conditions, re-exposure to a conditioned stimulus causes co-activation of the sympathetic and parasympathetic branches of the autonomic nervous system. Blockade of sympathetic outflow with propranolol decreases fear-associated tachycardia following auditory fear conditioning, while atropine enhances it (Iwata and LeDoux, 1988). Similar results have also been reported in contextual models of fear conditioning (Carrive, 2006). These findings suggest that the conditioned cardiovascular response consists of activation of the sympathetic nervous system (SNS), which is partially buffered by simultaneous activation of the parasympathetic nervous system (PSNS). Through cardiac nerves and circulating adrenal catecholamines, sympathetic activation results in an increased HR. Mean arterial pressure (MAP) also increases in response to sympathetically mediated blood vessel constriction (Baudrie et al., 2001). Parasympathetic activation, on the other hand, simultaneously acts to lower HR via cholinergic modulation of sinoatrial node activity. Given that blood pressure and HR are under autonomic regulation, but with distinct temporal and network control (Tovote et al., 2005a), both parameters should be considered during assessment of fear expression and extinction recall. To date, the effects of extinction training on conditioned blood pressure responses have not been directly tested.

Here we developed a novel conditioned cardiovascular response behavioral paradigm to examine the effects of extinction training on cue-dependent blood pressure and HR responses. We hypothesized that a fear conditioned cardiovascular response could be detected in a novel context and attenuated by extinction training. Closely evaluating cardiovascular reactivity to conditioned fear may contribute to a better understanding of the hyper-physiological responses in PTSD and associated CVD risk. Physiological measures of inhibitory learning could also lead to more accurate assessments of extinction efficiency in animal models. The objectives of this study were to examine the real-time behavioral and cardiovascular responses to fear conditioning and extinction using a mouse model, and to examine the effects of extinction training on MAP and HR responses during extinction recall.

## Materials and Methods

### Animals

Adult male (3–4 months old) C57BL/6J mice from Jackson Laboratory (Bar Harbor, ME, United States) were used for all experiments. The C57BL/6 strain is a commonly used inbred strain that has been shown to extinguish fear responses well in comparison to other strains (Hefner et al., 2008; Camp et al., 2012). Mice were housed individually in temperature and humidity-controlled polyethylene cages on a 12 h light/dark cycle. Animals were supplied with water and food *ad libitum* for the duration of the experiments. All procedures were approved by the Institutional Animal Care and Use Committee at The George Washington University and were in compliance with National Institutes of Health guidelines.

### Radiotelemetry

#### Telemeter Implantation, Data Collection and Analysis

Animals were anesthetized with an IP injection of ketamine/xylazine and maintenance of anesthesia was assessed with toe pinch. HDX-11 transmitters [Data Sciences International (DSI), St. Paul, MN, United States] were implanted subcutaneously, with a blood pressure transducer inserted into the carotid artery. Animals were allowed to recover for 14 days before beginning behavioral experiments. Blood pressure signals were sampled at a rate of 500 Hz. Blood pressure and activity data were continuously collected during 24 h baseline measurements, fear conditioning, extinction training, and cardiovascular response tests. Blood pressure data were analyzed using Ponemah software version 6.3 (DSI). Baseline day, night, and 24 h averages were calculated from 12 h epochs corresponding with the light/dark cycle. HR was derived from the blood pressure channel.

### Behavioral Experiments

#### Fear Conditioning

For 2 days prior to fear conditioning, animals were exposed to the chamber to habituate them to handling and context. Auditory fear conditioning was performed in conditioning test cages (7″ × 7″D12″H; model H10-11M-TC) equipped with overhead cameras and grid shock floors (H10-11M-TC-SF). Test cages were enclosed in sound attenuating isolation cubicles (Model H10-24T; Coulbourn Instruments, Holliston, MA, United States). Fear conditioned animals received both the conditioned stimulus and unconditioned stimulus (CS-US group), and were presented with CS-US pairings of a 30 s auditory cue (6 kHz, 75 db) co-terminating with a mild footshock (0.5 s, 0.5 mA). There was a 3 min 30 s inter-trial interval between each pairing. Control (CS group) animals were exposed to the CS under fear conditioning conditions but never received a footshock. Fear conditioning test cages were cleaned thoroughly with 70% ethanol before each session. After conditioning, animals were returned to the home cage for 24 h before extinction training.

#### Extinction Training

**FIGURE 1:**
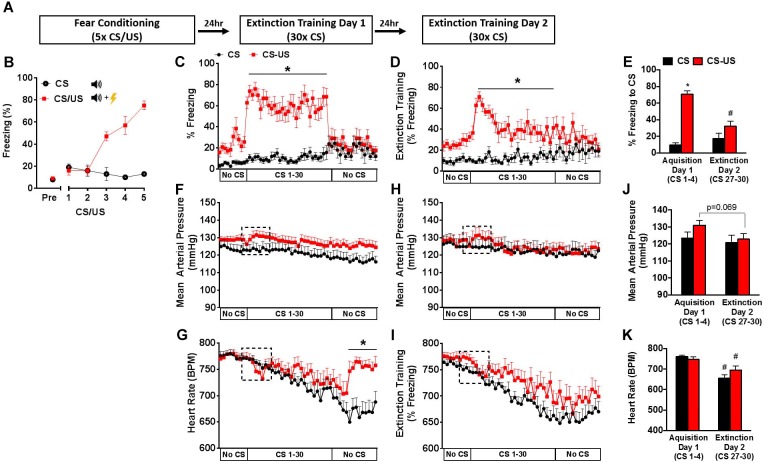
Behavioral and cardiovascular changes during extinction training. Schematic of fear conditioning and 2-day extinction protocol **(A)**. Freezing behavior during fear conditioning **(B)**. Freezing behavior **(C,D)**, mean arterial pressure **(F,H)**, and heart rate **(G,I)** during 2-day extinction training protocol. Average freezing behavior **(E)**, mean arterial pressure **(J)**, and heart rate **(K)** during CS 1–4 (Day 1), and CS 27–30 (Day 2) in the extinction context. Error bars indicate standard error of the mean (*n* = 9–11 per group, ^∗^*p* < 0.05 CS vs. CS-US; ^#^*p* < 0.05 Day 1 vs. Day 2).

Two rounds of extinction training were performed in modified test cages to distinguish them from the fear conditioning context. The shock grid was replaced with a clear plexiglass floor and the clear chamber walls were covered with paper. The chambers were wiped down with water and peppermint soap before each extinction session. Extinction training occurred 24 and 48-h following fear conditioning (Figure 1A). A 5 min pre-CS period preceded the first tone presentation in each test. Extinction sessions consisted of either 30 conditioned stimulus tone trials in CS and CS-US groups, or 35 trials in the Extinction (Ext) group. Each trial lasted 30 s and was followed by a 30 s inter-trial interval. The No Extinction (No Ext) control group was placed into the modified context for the same duration, but was not exposed to the conditioned stimulus during extinction training. A non-conditioned (No US) control group was not included in the home cage extinction experiments based on previous studies showing that the auditory stimulus would evoke only mild, transient cardiovascular effects that do not differ significantly from baseline values (Tovote et al., 2005a). The percentage of time spent freezing was calculated using Freezeframe 3.32 (Coulbourn Instruments). All behavioral experiments occurred during the light phase (7am–7pm).

#### Cardiovascular Response Tests

**FIGURE 2:**
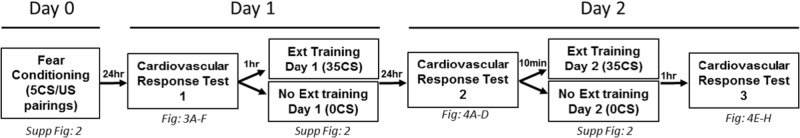
Schematic of fear conditioning and testing protocol for home cage extinction studies. All animals were fear conditioned on Day 0. Conditioned cardiovascular responses were measured in the home cage 24 h later (Cardiovascular Response Test 1). Animals were then placed into a novel context and exposed to either 35 CS (Ext group) or no CS (No Ext group) for the first extinction session. On Day 2, a home cage test (Cardiovascular Response Test 2) was conducted prior to the second extinction session. The Ext group was then exposed to another 35CS extinction session. One hour later, all animals were tested in the home cage (Cardiovascular Response Test 3).

The conditioned cardiovascular response was measured in the home cage 24-h after fear conditioning (Cardiovascular Response Test 1), 24-h after the first extinction session (Cardiovascular Response Test 2), and 1-h after the second extinction session (Cardiovascular Response Test 3) (Figure 2). The home cage was placed in a sound attenuating chamber, and a speaker was positioned on top of the cage. Mice were left undisturbed for 1 h before remotely initiating a 4 CS memory test to determine the effects of extinction training on the conditioned cardiovascular response during extinction recall.

### Statistical Analysis

Prism 6.0 (Graphpad Software Inc., La Jolla, CA, United States) was used for statistical evaluation of mouse data. Data are presented as the mean ± SEM, with *p-*values <0.05 considered statistically significant. Analysis of variance (ANOVA) for repeated measures was used for statistical analysis followed by Bonferroni tests for *post hoc* comparison.

## Results

### Behavioral and Cardiovascular Responses During Extinction Training in a Novel Context

To examine the cardiovascular responses to conditioned fear during extinction training, two groups of animals were equipped with radiotelemeters and were either exposed to a fear conditioning protocol (CS-US group) or exposed to five auditory cues without footshocks (CS group). Behavioral and cardiovascular responses (freezing behavior, MAP, and HR) were simultaneously monitored in the extinction context during two consecutive days of extinction training in the CS-US and CS groups (Figure 1A).

#### Freezing Behavior

As expected, a progressive increase in freezing response to CS exposure was observed only in the CS-US animals (Figure 1B). Simultaneous behavioral and cardiovascular responses to extinction trials across 2 days are shown in Figures 1C–K. The average of the first 4 CS (CS1–4) presentations on Day 1 and the last 4 CS presentations on Day 2 (CS27–30) were taken as measures of acquisition and extinction, respectively (Yang et al., 2016). On Day 1 of extinction training, the CS-US group exhibited increased freezing throughout the 30 CS presentations (Figure 1C). Fear acquisition was demonstrated in the CS-US animals compared to the CS by group differences in freezing during the first 4 CS presentations (71% ± 4 vs. 10% ± 3, *p* < 0.05) (Figure 1E). CS-US animals showed a significant reduction in freezing from Day 1–2, with a significant group x time interaction [*F*(1,17) = 20.68, *p* = 0.0003] (Figure 1E).

#### Mean Arterial Pressure and HR in Novel Context

At baseline on Day 1 of extinction training, MAP and HR were similar between groups (Figures 1F,G). In response to the first CS, a trend for a small increase in MAP (3 mmHg), which remained elevated throughout the extinction session and a corresponding bradycardic HR response were observed in the CS-US group compared to control. Overall, both groups showed a slow reduction in HR throughout the extinction session. Following the last CS presentation, the CS-US group displayed a rapid increase in HR corresponding with the cessation of freezing. There was a significant group by time interaction [*F*(48,864) = 5.032, *p* < 0.0001], with group differences in HR throughout the No CS period (Figure 1G).

On Day 2 of extinction training, in response to CS, there was a similar trend for a small transient increase in MAP and bradycardic response within the CS-US animals only during CS 1–4. For the remainder of the session, MAP was similar between groups while HR slowly declined (-100 bpm). Unlike Day 1 of extinction, the CS-US group did not exhibit the sharp increase in HR during the No CS period. MAP responses were then evaluated across days between extinction sessions (Figure 1J). When comparing the first 4 CSs (Day 1 of extinction) and the last 4 CSs (Day 2 of extinction), repeated measures-ANOVA revealed a main effect of time, [*F*(1,18) = 14.28, *p* = 0.0014], and a trend for group by time interaction [*F*(1,18) = 3.736, *p* = 0.0691]. There was also a reduction in HR in response to CS from Day 1 of extinction to Day 2 of extinction in CS-US animals (Figure 1K). However, because a comparison of HR between groups revealed a significant group by time interaction [*F*(1,18) = 5.936, *p* = 0.0254] with a main effect of time [*F*(1,18) = 52.52, *p* < 0.0001] but not group [*F*(1,18) = 0.3778, *p* = 0.5465], this reduction cannot be attributed solely to extinction learning and is more likely a result of within session HR recovery.

In addition, both groups displayed a slow, gradual recovery of HR throughout each session, but neither MAP nor HR returned to resting baseline levels by the end of the test on either day. This suggests that handling and novel context exposure contribute to the elevations of MAP and HR regardless of whether or not the animals were fear conditioned. Furthermore, because these HR elevations are similar at the beginning of both days of extinction training, the effects of habituation appear to be minimal. These findings are consistent with previous studies showing that novel environments can induce HR elevations in mice despite previous habituation (Liu et al., 2013). Because elevated baseline cardiovascular measures could potentially mask cardiovascular adjustments caused by the conditioned stimulus, we next examined the effects of extinction training on fear conditioned (CS-US) mouse cardiovascular reactivity in a home cage environment.

### CS-Dependent Conditioned Cardiovascular Responses in the Home Cage

#### Mean Arterial Pressure and HR Response (Cardiovascular Response Test 1)

**FIGURE 3:**
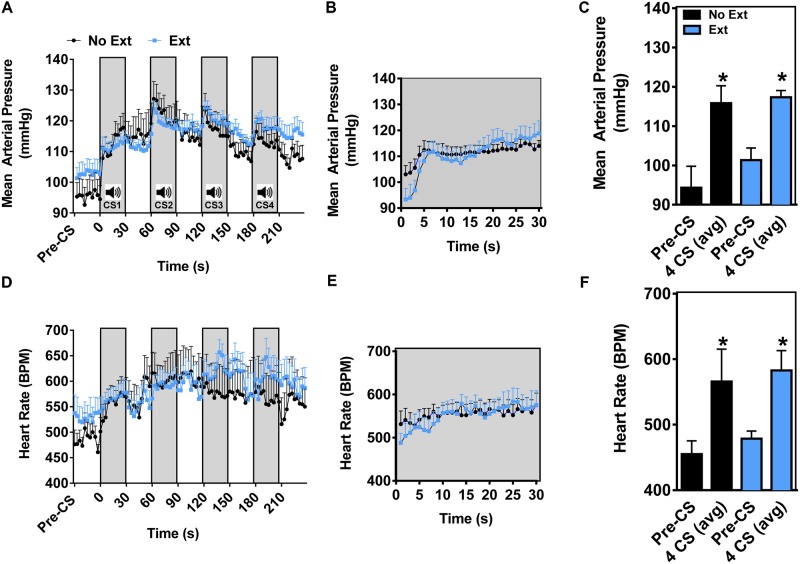
Pre-extinction training CS-dependent cardiovascular response test 1 (home cage). Mean arterial pressure **(A)**, and heart rate **(D)** collected during 4 CS presentations and averaged every 3 s. **(B,E)** Depict second-by-second fluctuations in MAP and HR during the first CS presentation. Average MAP **(C)** and HR **(F)** during the 5 min pre-CS period, and over 4 CS presentations (*n* = 6–9 per group, ^∗^*p* < 0.05 pre-CS vs. 4 CS avg).

Conditioned physiological responses are highly dependent on the resting physiological state, which in part influences the cardiovascular response to conditioned stimuli. Therefore, in order to examine both the conditioned cardiovascular responses and the effects of extinction, cardiovascular response tests were conducted in the home cage environment (Stiedl et al., 2004). Two groups of mice (No Ext and Ext) were equipped with radiotelemeters and all animals were fear conditioned as previously described (Figure 2). 24 h after fear conditioning (prior to extinction training), mice were exposed to 4 CS trials in the home cage (Cardiovascular Response Test 1). Baseline activity levels, blood pressure and HR during the pre-CS period were significantly lower than in the extinction context in both groups (Supplementary Figure S1), while Pre-CS cardiovascular baselines were similar to mean 12-h baselines during the light cycle (Supplementary Table S1). As shown in Figure 3A, a two-phase pressor response was observed during the first CS presentation of the 4 CS test in both groups. This consisted of a rapid rise (general arousal) of approximately 10 mmHg within the first 10 s of the CS, followed by a slower, steady increase which has previously been attributed to associative learning (Tovote et al., 2005a; Figure 3B). Subsequent CS presentations also coincided with a rapid rise in MAP. Peak MAP (No Ext 122 ± 5; Ext 124 ± 3 mmHg) were reached within the first 3 s of the second CS. MAP averaged over the 4 CSs was significantly increased from Pre-CS baselines in both No Ext and Ext groups confirming a strong conditioned CS-dependent MAP pressor response in these animals (Figure 3C). An ANOVA comparing the 4 CS MAP revealed no significant main effect of group [*F*(1,13) = 1.212, *p* = 0.2908] or group by time interaction [*F*(1,13) = 0.7181, *p* = 0.4121], yet did reveal a significant main effect of time [*F*(1,13) = 33.38, *p* < 0.0001]. As shown in Figures 3D–F, there was an overall significant increase (∼100 bpm) in HR over the 4 CS trials in both groups relative to pre-CS baseline. An ANOVA revealed a main effect of time [*F*(1,11) = 10.59, *p* = 0.0077], with no group by time interaction [*F*(1,11) = 0.01081, *p* = 0.9190] and no main effect of group [*F*(1,11) = 0.6541, *p* = 0.4358]. In summary, these data demonstrate a consistent CS-dependent home cage pressor response that was accompanied by an overall increase in HR 24 h following fear conditioning.

### Extinction of CS-Dependent Cardiovascular Responses

#### Mean Arterial Pressure and HR Response (Cardiovascular Response Test 2)

**FIGURE 4:**
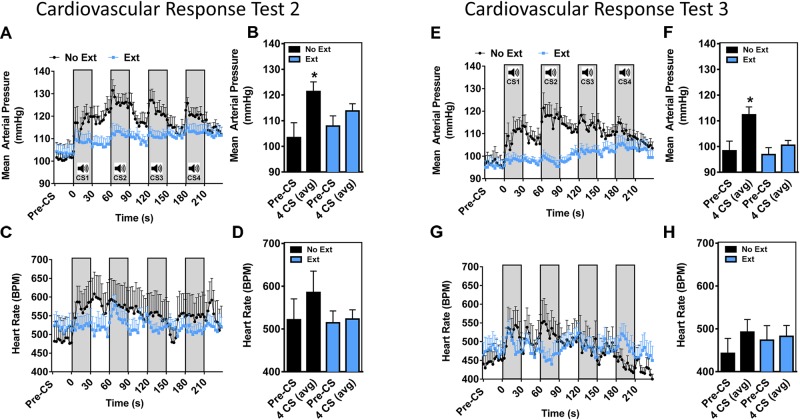
Post-extinction training CS-dependent cardiovascular response tests 2 and 3 (home cage). Mean arterial pressure **(A)** and heart rate **(C)** collected during cardiovascular response test 2. Average MAP **(B)** and HR **(D)** during 4 CS presentations. Mean arterial pressure **(E)**, and heart rate **(G)** collected during cardiovascular response test 3. Average MAP **(F)** and HR **(H)** during 4 CS presentations (*n* = 6–9 per group, ^∗^*p* < 0.05 pre-CS vs. 4 CS avg).

The two groups of animals subsequently went through either an Ext or No Ext training protocol as shown in Figure 2. Following extinction training, animals were tested at two time points in order to examine (A) long-term retention of the CS-dependent cardiovascular response and (B) within-session cumulative effects of additional extinction trials. To examine the long-term retention of extinction learning during the CS-dependent cardiovascular response, a 4-tone cardiovascular response test (#2) was conducted 24 h following extinction training. As shown in Figures 4A,C, 5 min pre-CS baseline MAP and HR were similar between Ext and No Ext groups (MAP: 103 ± 6 vs. 108 ± 4 mmHg; HR: 522 ± 49 vs. 515 ± 28 bpm) and within the range of normal daytime averages (Supplementary Table S1). Similar to the initial CS response in Figure 3A, the No Ext group displayed a biphasic pressure increase that was characterized by a rapid increase in MAP (∼10 mmHg) within the first 10 s, followed by a slower increase that persisted until the end of the first CS. In these animals, peak MAP (132 mmHg) was again reached during the first 3 s of the second CS presentation (Figure 4A). An ANOVA comparing the 4 CS MAP revealed a significant group by time interaction [*F*(1,13) = 5.164, *p* = 0.0407] (Figure 4B). *Post hoc* tests revealed that MAP in the No-Ext group significantly increased from baseline, while MAP in the Ext group did not. In animals that underwent extinction training (Ext group), the initial rise in blood pressure of ∼10 mmHg was observed, however, the second phase was distinctively absent (Figure 4A). Despite a trend for a change in HR increase to CS in the No Ext group (Figure 4C), HR changes were highly variable (Figure 4C). An ANOVA comparing the 4 CS HR revealed no significant group by time interaction [*F*(1,13) = 2.463, *p* = 0.1406] (Figure 4D). These results demonstrate that 24 h after training, the conditioned MAP response is significantly blunted in the Ext group while the HR response to CS is not significantly different between groups, and thus may track closely to the extinguished freezing behavior.

#### Mean Arterial Pressure and HR Response (Cardiovascular Response Test 3)

To further evaluate the effects of extinction on the conditioned cardiovascular response, the Ext group was exposed to a second day of extinction training, which resulted in an extinction effect as determined by freezing responses to CS (Supplementary Figure S2). All animals were tested 1 h later in their home cage for CS-dependent cardiovascular responses. Consistent with the two previous cardiovascular response tests, a two-phase blood pressure response was observed in the No Ext animals and the peak MAP (122 ± 7) was reached within the first 3 s of the second CS (Figure 4E). In the Ext group a small, slow increase in MAP was observed throughout the test (∼5 mmHg) but this increase did not appear to be associated with CS onset. Peak MAP in the Ext group was significantly lower than that of the No Ext group (106 mmHg ± 3 vs. 122 ± 7, *p* < 0.05) (Figure 4E). Moreover, both phases of the MAP response were absent in the Ext animals. An ANOVA comparing the 4 CS MAP revealed a significant group by time interaction [*F*(2,16) = 4.409, *p* = 0.0298] (Figure 4F). Similar comparisons of HR indicate no group by time interaction [*F*(1,12) = 1.504, *p* = 0.2436] (Figure 4H). Peak HR were not significantly different between No Ext (555 ± 61) and Ext (523 ± 28) groups (Figure 4G) and remained within the range of normal resting HRs (Supplementary Table S1).

## Discussion

The current findings demonstrate that recall of consolidated extinction memories can modulate the conditioned cardiovascular response, which is influenced by context-dependent differences in blood pressure and HR sensitivity. Alterations of the conditioned MAP response may serve as a novel index in the evaluation of extinction efficiency and may aid in identifying the hyper-physiological underpinnings of PTSD and co-morbid CVD-PTSD.

Conditioned HR (Fitzgerald and Martin, 1971) and BP (Iwata and LeDoux, 1988) during CS presentation have previously been reported in the conditioning context, which elicits contextual and non-specific stress related alterations in cardiovascular activity. Furthermore, freezing behavior and locomotor activity are closely linked to changes in blood pressure and HR (Vliet et al., 2003) and can potentially confound conditioned cardiovascular responses. To minimize these disruptions, conditioned cardiovascular responses are often evaluated in the resting or home cage environment (Iwata and LeDoux, 1988; Stiedl et al., 2004, 2009; Camp et al., 2012). In the present study, we predicted that testing in a novel context would reduce contextual fear and minimize non-specific stress enough to observe HR and MAP responses to CS between conditioned (CS-US) and control (CS) groups. Additionally, we hypothesized that blood pressure and HR responses in the extinction context would decrease across days as a result of extinction training.

During the first extinction session in a novel context (Day 1), freezing in response to CS confirmed fear acquisition in conditioned animals, while the magnitude of the cardiovascular response was not significantly different from controls. Throughout the CS period on Days 1 and 2 of extinction, MAP remained high while HR gradually fell in both groups over the CS period, indicating a within-session habituation effect on HR but not MAP. While the relatively small conditioned cardiovascular response limits the interpretation of these findings, it is possible that the small elevation of MAP in the CS-US group on Day 1 was sustained by repeated CS presentation and extinguished by repeated CS exposure, while HR increases were offset by reductions in physical activity during times of freezing.

During the second extinction session in a novel context (Day 2), there was trend for a reduction in MAP in the CS-US group only, while HR was significantly reduced in both groups. These data may suggest an extinction-specific reduction of blood pressure response that was distinct from a generalized habituation-like effect on HR. Interestingly, following cessation of CS presentation during extinction training on Day 1, CS-US animals displayed a rapid increase in HR. While this may have been a consequence of increased locomotion due to an immediate reduction in freezing, there were no overall differences in activity levels between groups during this time period. Because conditioned HR responses are controlled by activation of both the sympathetic and parasympathetic components of the autonomic nervous system (Iwata and LeDoux, 1988; Carrive, 2006), and freezing behavior is accompanied by parasympathetically driven HR deceleration (Roelofs, 2017), this increase in HR may be mediated in part by parasympathetic withdrawal. Similarly, on both day 1 and 2 in the novel context we observed a initial brief decelerative HR response upon CS onset (Figures 1G,I), which is likely parasympathetically mediated (Iwata and LeDoux, 1988). This deceleration in HR is consistent with prior human fear research using HR (typically associated with fear responding) (Lang et al., 2011; Sege et al., 2017). Taken together, these findings suggest that CS exposure in a novel context may elicit MAP and HR responses at different rates, with changes in autonomic regulation of HR first emerging upon CS onset (deceleration) and again when the conditioned stressor is removed (acceleration).

To further investigate extinction-dependent responses in our mouse model, while minimizing context-enhanced basal cardiovascular effects (see Supplementary Figure S1), we next sought to determine whether repeated CS exposure could extinguish conditioned cardiovascular responses when measured in the home cage. As opposed to previous studies using an extended single CS (Stiedl, 1999; Stiedl et al., 2004, 2007, 2009; Tovote et al., 2005a), we evaluated the cardiovascular response using 4 CS presentations. A 4 CS memory test was used based on the following considerations: (1) the cumulative effect of multiple CS presentations could be determined by using shorter CS presentations spaced with inter-trial intervals; (2) the test duration would be long enough to encompass both the fast, sympathetic-mediated vasoconstriction response (Baudrie et al., 1997), and the slower humoral-mediated responses (Tovote et al., 2005a); (3) the low number of CS presentations would minimize extinction in the No Ext group caused by multiple testing sessions.

In the home cage context, both groups of mice showed stable resting MAP and HR during the pre-CS period. Prior to extinction training and 24-h post-fear conditioning, MAP increased across the 4 CS trials in a CS-dependent manner. This increase reached ∼20 mm Hg and was characterized by two phases of rise. The initial, rapid increase is thought to result from general arousal and lasts for approximately 5 s. The second phase of the conditioned blood pressure response, which has been shown to result from associative fear learning in mice, allows for distinction between general arousal and associative memory (Tovote et al., 2005a). While HR was increased in both groups across CS trials, the magnitude of these responses was markedly less than previously described (Stiedl and Spiess, 1997). Variations in conditioning protocols and testing procedures likely account for these differences (Stiedl and Spiess, 1997; Stiedl, 1999; Stiedl et al., 2004, 2007; Tovote et al., 2005a). Based on the clear presence of an associative blood pressure response in this experiment, and the ability of a 30 CS extinction protocol to reduce long-term fear expression (Marvar et al., 2014), we reasoned that the MAP response would be suppressed during extinction recall.

To evaluate the cardiovascular response during long-term extinction memory recall, animals were re-tested 24 h after extinction training in the home cage. The No Ext group exhibited a CS-dependent MAP increase across 4 CS home cage trials, while extinguished animals did not. Furthermore, the general arousal phase of the MAP response was present, while the associative phase was absent in the Ext group. These data suggest that extinguished animals respond with an acute, generalized arousal similar to non-extinguished animals, while the associative component of the blood pressure response can be modified by non-reinforced CS exposure.

Despite the extinction-dependent reduction in MAP response, there was no significant difference in HR response between groups. Although we observed a trend for increased HR in the No Ext across the 4 CS presentations, it was not significantly different from the pre-CS period at this time point. We attribute the lack of a conditioned HR response to the increased variability and small conditioned HR response observed in this model. Either the fear conditioning protocol did not result in a strong enough conditioned HR response to detect the effects of extinction between Ext and No Ext groups, or the HR response was so sensitive to CS presentation in the home cage that it was quickly extinguished in both groups following the first home cage test. In either case, the results of this study point to MAP as a reliable index of extinction in mouse models of fear conditioning. Taken together, the MAP and HR data from these experiments show that that the conditioned blood pressure response is significantly blunted by extinction training while the HR response did not allow for distinction between extinguished and non-extinguished control animals.

Short-term effects of an additional extinction training session were also evaluated during the final home cage test. Consistent with the two previous cardiovascular response tests, a two-phase blood pressure response was observed in the No Ext animals with peak MAP values occurring within the first 3 s of the second CS (Figure 4E). There were no significant HR responses in either group at this timepoint. The results of this extinction test confirm a reduction in the MAP response in the Ext group and show minimal change of HR in response to CS in either group. Interestingly, conditioned HR responses in our mouse model in the home cage were not as robust as those previously reported by other groups (Stiedl and Spiess, 1997; Tovote et al., 2005a). As a result, HR increases in resting animals were seemingly extinguished during home cage testing. Future studies will need to address the effects of conditioned stimulus intensity and duration on HR responses.

In summary, the present study demonstrates for the first time that extinction training attenuates the acute blood pressure responses evoked by conditioned stimuli and that in this behavioral paradigm, MAP was a more reliable measure of conditioning and extinction than HR. Moreover, this reduction of the fear-induced cardiovascular response appears to be independent of activity-related behavioral changes. While there is some evidence to suggest that extinction training can attenuate cardiac responses in humans (Panitz et al., 2015), future studies are required to determine the impact of extinction-based therapeutic interventions on cardiovascular reactivity in PTSD patients. Such studies may potentially lead to improvements in extinction-based therapies and PTSD–CVD comorbidity outcomes.

## Author Contributions

PM and APS contributed to the conception of the work, data analysis, results interpretation, drafting, revision and final approval of the article. AVS and JP contributed to data analysis, results interpretation, revision and final approval of the article.

## Conflict of Interest Statement

The authors declare that the research was conducted in the absence of any commercial or financial relationships that could be construed as a potential conflict of interest.

## Supplementary Material

FIGURE S1 Baseline cardiovascular measures in training and home cage contexts. Activity level **(A)**, mean arterial pressure **(B)**, diastolic pressure **(C)**, systolic pressure **(D)**, and heart rate **(E)** over the 5 min pre-CS period in each context (*n* = 9–11 per group. ^∗^*p* < 0.05 Training vs. Home Cage).

FIGURE S2 Freezing behavior during fear conditioning **(A)**, extinction training Day 1 **(B)**, and extinction training Day 2 **(C)**. All animals received a footshock and CS during fear conditioning (*n* = 6–9 per group).

TABLE S1 Baseline day/night (12 h) mean arterial pressure (MAP), heart rate (HR), and activity levels in mice prior to fear conditioning.

## References

[B1] American Psychiatric Association (2013). *Diagnostic and Statistical Manual of Mental Disorders*, 5th Edn. Virginia, NV: American Psychiatric Association 10.1176/appi.books.9780890425596

[B2] Baudrie V. Laude D. Chaouloff F. Elghozi J.-L. (2001). Genetic influences on cardiovascular responses to an acoustic startle stimulus in rats. *Clin. Exp. Pharmacol. Physiol.* 28 1096–1099. 10.1046/j.1440-1681.2001.03593.x 11903324

[B3] Baudrie V. Tulen J. H. Blanc J. Elghozi J. L. (1997). Autonomic components of the cardiovascular responses to an acoustic startle stimulus in rats. *J. Auton. Pharmacol.* 17 303–309. 10.1046/j.1365-2680.1997.00465. 9427109

[B4] Bowers M. E. Ressler K. J. (2015). An overview of translationally informed treatments for PTSD: animal models of pavlovian fear conditioning to human clinical trials. *Biol. Psychiatry* 78 E15–E27. 10.1016/j.biopsych.2015.06.008 26238379 PMC4527085

[B5] Bukalo O. Pinard C. R. Holmes A. (2014). Mechanisms to medicines: elucidating neural and molecular substrates of fear extinction to identify novel treatments for anxiety disorders. *Br. J. Pharmacol.* 171 4690–4718. 10.1111/bph.12779 24835117 PMC4209938

[B6] Camp M. C. Macpherson K. P. Lederle L. Graybeal C. Gaburro S. Debrouse L. M. (2012). Genetic strain differences in learned fear inhibition associated with variation in neuroendocrine, autonomic, and amygdala dendritic phenotypes. *Neuropsychopharmacology* 37 1534–1547. 10.1038/npp.2011.340 22334122 PMC3327858

[B7] Carrive P. (2006). Dual activation of cardiac sympathetic and parasympathetic components during conditioned fear to context in the rat. *Clin. Exp. Pharmacol. Physiol.* 33 1251–1254. 10.1111/j.1440-1681.2006.04519.x 17184510

[B8] Desmedt A. Marighetto A. Piazza P. V. (2015). Abnormal fear memory as a model for posttraumatic stress disorder. *Biol. Psychiatry* 78 290–297. 10.1016/j.biopsych.2015.06.017 26238378

[B9] Edmondson D. Kronish I. M. Shaffer J. A. Falzon L. Burg M. M. (2013). Posttraumatic stress disorder and risk for coronary heart disease: a meta-analytic review. *Am. Heart J.* 166 806–814. 10.1016/j.ahj.2013.07.031 24176435 PMC3815706

[B10] Edmondson D. von Känel R. (2017). Post-traumatic stress disorder and cardiovascular disease. *Lancet Psychiatry* 4 320–329. 10.1016/S2215-0366(16)30377-7 28109646 PMC5499153

[B11] Fitzgerald R. D. Martin G. K. (1971). Heart-rate conditioning in rats as a function of interstimulus interval. *Psychol. Rep.* 29 1103–1110. 10.2466/pr0.1971.29.3f.1103 5139347

[B12] Gaburro S. Stiedl O. Giusti P. Sartori S. B. Landgraf R. Singewald N. (2011). A mouse model of high trait anxiety shows reduced heart rate variability that can be reversed by anxiolytic drug treatment. *Int. J. Neuropsychopharmacol.* 14 1341–1355. 10.1017/S1461145711000058 21320392 PMC3198175

[B13] Hager T. Maroteaux G. du Pont P. Julsing J. van Vliet R. Stiedl O. (2014). Munc18-1 haploinsufficiency results in enhanced anxiety-like behavior as determined by heart rate responses in mice. *Behav. Brain Res.* 260 44–52. 10.1016/j.bbr.2013.11.033 24304718

[B14] Hefner K. Whittle N. Juhasz J. Norcross M. Karlsson R. M. Saksida L. M. (2008). Impaired fear extinction learning and cortico-amygdala circuit abnormalities in a common genetic mouse strain. *J. Neurosci.* 28 8074–8085. 10.1523/JNEUROSCI.4904-07.2008 18685032 PMC2547848

[B15] Hsu Y. C. Yu L. Chen H. Lee H. L. Kuo Y. M. Jen C. J. (2012). Blood pressure variations real-time reflect the conditioned fear learning and memory. *PLoS One* 7:e32855. 10.1371/journal.pone.0032855 22496737 PMC3319555

[B16] Iwata J. LeDoux J. E. (1988). Dissociation of associative and nonassociative concomitants of classical fear conditioning in the freely behaving rat. *Behav. Neurosci.* 102 66–76. 10.1037/0735-7044.102.1.66 3355660

[B17] Jovanovic T. Norrholm S. D. Blanding N. Q. Davis M. Duncan E. Bradley B. (2010). Impaired fear inhibition is a biomarker of PTSD but not depression. *Depress. Anxiety* 27 244–251. 10.1002/da.20663 20143428 PMC2841213

[B18] Lang P. J. Wangelin B. C. Bradley M. M. Versace F. Davenport P. W. Costa V. D. (2011). Threat of suffocation and defensive reflex activation: threat of suffocation. *Psychophysiology* 48 393–396. 10.1111/j.1469-8986.2010.01076.x 20667037 PMC3620017

[B19] Liu J. Wei W. Kuang H. Zhao F. Tsien J. Z. (2013). Changes in heart rate variability are associated with expression of short-term and long-term contextual and cued fear memories. *PLoS One* 8:e63590. 10.1371/journal.pone.0063590 23667644 PMC3646801

[B20] Marvar P. J. Goodman J. Fuchs S. Choi D. C. Banerjee S. Ressler K. J. (2014). Angiotensin type 1 receptor inhibition enhances the extinction of fear memory. *Biol. Psychiatry* 75 864–872. 10.1016/j.biopsych.2013.08.024 24094510 PMC3975818

[B21] Milad M. R. Pitman R. K. Ellis C. B. Gold A. L. Shin L. M. Lasko N. B. (2009). Neurobiological basis of failure to recall extinction memory in posttraumatic stress disorder. *Biol. Psychiatry* 66 1075–1082. 10.1016/j.biopsych.2009.06.026 19748076 PMC2787650

[B22] Morrison F. G. Ressler K. J. (2014). From the neurobiology of extinction to improved clinical treatments. *Depress. Anxiety* 31 279–290. 10.1002/da.22214 24254958 PMC4293038

[B23] Myers B. (2017). Corticolimbic regulation of cardiovascular responses to stress. *Physiol. Behav.* 172 49–59. 10.1016/j.physbeh.2016.10.015 27793557 PMC5618801

[B24] Panitz C. Hermann C. Mueller E. M. (2015). Conditioned and extinguished fear modulate functional corticocardiac coupling in humans. *Psychophysiology* 52 1351–1360. 10.1111/psyp.12498 26189975

[B25] Park J. Marvar P. J. Liao P. Kankam M. L. Norrholm S. D. Downey R. M. (2017). Baroreflex dysfunction and augmented sympathetic nerve responses during mental stress in veterans with post-traumatic stress disorder. *J. Physiol.* 595 4893–4908. 10.1113/JP274269 28503726 PMC5509856

[B26] Quirk G. J. (2002). Memory for extinction of conditioned fear is long-lasting and persists following spontaneous recovery. *Learn. Mem.* 9 402–407. 10.1101/lm.49602 12464700 PMC187587

[B27] Quirk G. J. Russo G. K. Barron J. L. Lebron K. (2000). The role of ventromedial prefrontal cortex in the recovery of extinguished fear. *J. Neurosci.* 20 6225–6231. 10.1523/JNEUROSCI.20-16-06225.2000 10934272 PMC6772571

[B28] Roelofs K. (2017). Freeze for action: neurobiological mechanisms in animal and human freezing. *Philos. Trans. R. Soc. Lond. B Biol. Sci.* 372:20160206. 10.1098/rstb.2016.0206 28242739 PMC5332864

[B29] Sege C. T. Bradley M. M. Lang P. J. (2017). Escaping aversive exposure. *Psychophysiology* 54 857–863. 10.1111/psyp.12842 28218794 PMC5423845

[B30] Sijbrandij M. Engelhard I. M. Lommen M. J. J. Leer A. Baas J. M. P. (2013). Impaired fear inhibition learning predicts the persistence of symptoms of posttraumatic stress disorder (PTSD). *J. Psychiatr. Res.* 47 1991–1997. 10.1016/j.jpsychires.2013.09.008 24090716

[B31] Stiedl O. (1999). Strain and substrain differences in context- and tone-dependent fear conditioning of inbred mice. *Behav. Brain Res.* 104 1–12. 10.1016/S0166-4328(99)00047-9 11125727

[B32] Stiedl O. Jansen R. F. Pieneman A. W. Ögren S. O. Meyer M. (2009). Assessing aversive emotional states through the heart in mice: implications for cardiovascular dysregulation in affective disorders. *Neurosci. Biobehav. Rev.* 33 181–190. 10.1016/j.neubiorev.2008.08.015 18824021

[B33] Stiedl O. Misane I. Koch M. Pattij T. Meyer M. Ögren S. O. (2007). Activation of the brain 5-HT2C receptors causes hypolocomotion without anxiogenic-like cardiovascular adjustments in mice. *Neuropharmacology* 52 949–957. 10.1016/j.neuropharm.2006.10.012 17141810

[B34] Stiedl O. Spiess J. (1997). Effect of tone-dependent fear conditioning on heart rate and behavior of C57BL/6N mice. *Behav. Neurosci.* 111 703–711. 10.1037/0735-7044.111.4.70 9267648

[B35] Stiedl O. Tovote P. Ögren S. O. Meyer M. (2004). Behavioral and autonomic dynamics during contextual fear conditioning in mice. *Auton. Neurosci.* 115 15–27. 10.1016/j.autneu.2004.07.006 15507402

[B36] Tovote P. Meyer M. Pilz P. K. D. Ronnenberg A. Ögren S. O. Spiess J. (2005a). Dissociation of temporal dynamics of heart rate and blood pressure responses elicited by conditioned fear but not acoustic startle. *Behav. Neurosci.* 119 55–65. 10.1037/0735-7044.119.1.55 15727512

[B37] Tovote P. Meyer M. Ronnenberg A. Ögren S. O. Spiess J. Stiedl O. (2005b). Heart rate dynamics and behavioral responses during acute emotional challenge in corticotropin-releasing factor receptor 1-deficient and corticotropin-releasing factor-overexpressing mice. *Neuroscience* 134 1113–1122. 10.1016/j.neuroscience.2005.05.027 16039799

[B38] Vaccarino V. Goldberg J. Rooks C. Shah A. J. Veledar E. Faber T. L. (2013). Post-traumatic stress disorder and incidence of coronary heart disease: a twin study. *J. Am. Coll. Cardiol.* 62 970–978. 10.1016/j.jacc.2013.04.085 23810885 PMC3823367

[B39] Vliet B. N. V. Chafe L. L. Montani J. P. (2003). Characteristics of 24 h telemetered blood pressure in eNOS-knockout and C57Bl/6J control mice. *J. Physiol.* 549 313–325. 10.1113/jphysiol.2003.041897 12665600 PMC2342911

[B40] Yang W. Z. Liu T. T. Cao J. W. Chen X. F. Liu X. Wang M. (2016). Fear erasure facilitated by immature inhibitory neuron transplantation. *Neuron* 92 1352–1367. 10.1016/j.neuron.2016.11.018 27939579

