# Supplementary figures and images for: Extinction of Fear Memory Attenuates Conditioned Cardiovascular Fear Reactivity
Source: Front Behav Neurosci. 2018 Nov 13;12:276. doi: 10.3389/fnbeh.2018.00276 (PMC6244092; doi:10.3389/fnbeh.2018.00276)

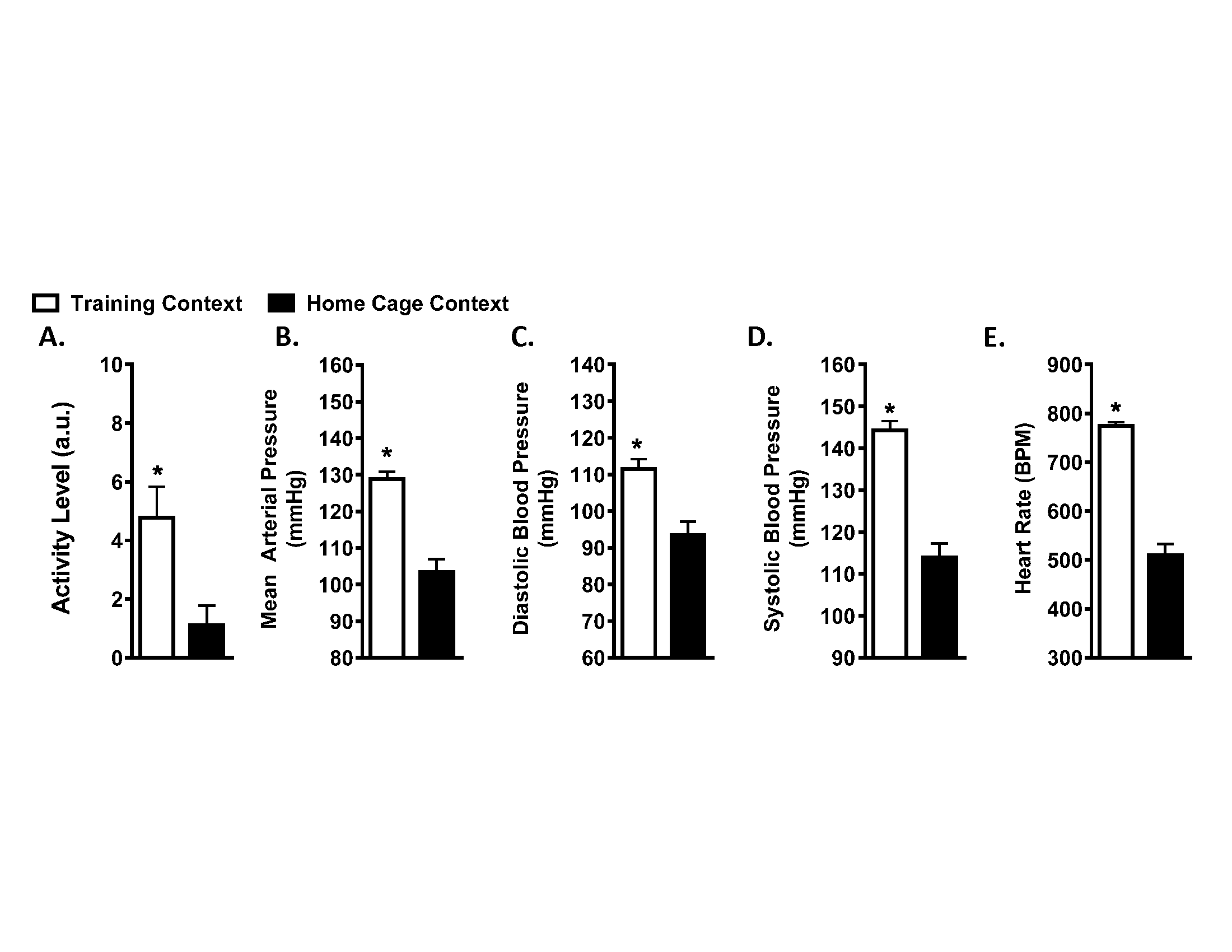

Supplement: FIGURE S1 — Baseline cardiovascular measures in training and home cage contexts. Activity level (A), mean arterial pressure (B), diastolic pressure (C), systolic pressure (D), and heart rate (E) over the 5 min pre-CS period in each context (n = 9–11 per group. ∗p < 0.05 Training vs. Home Cage). [file Image_1.tif]

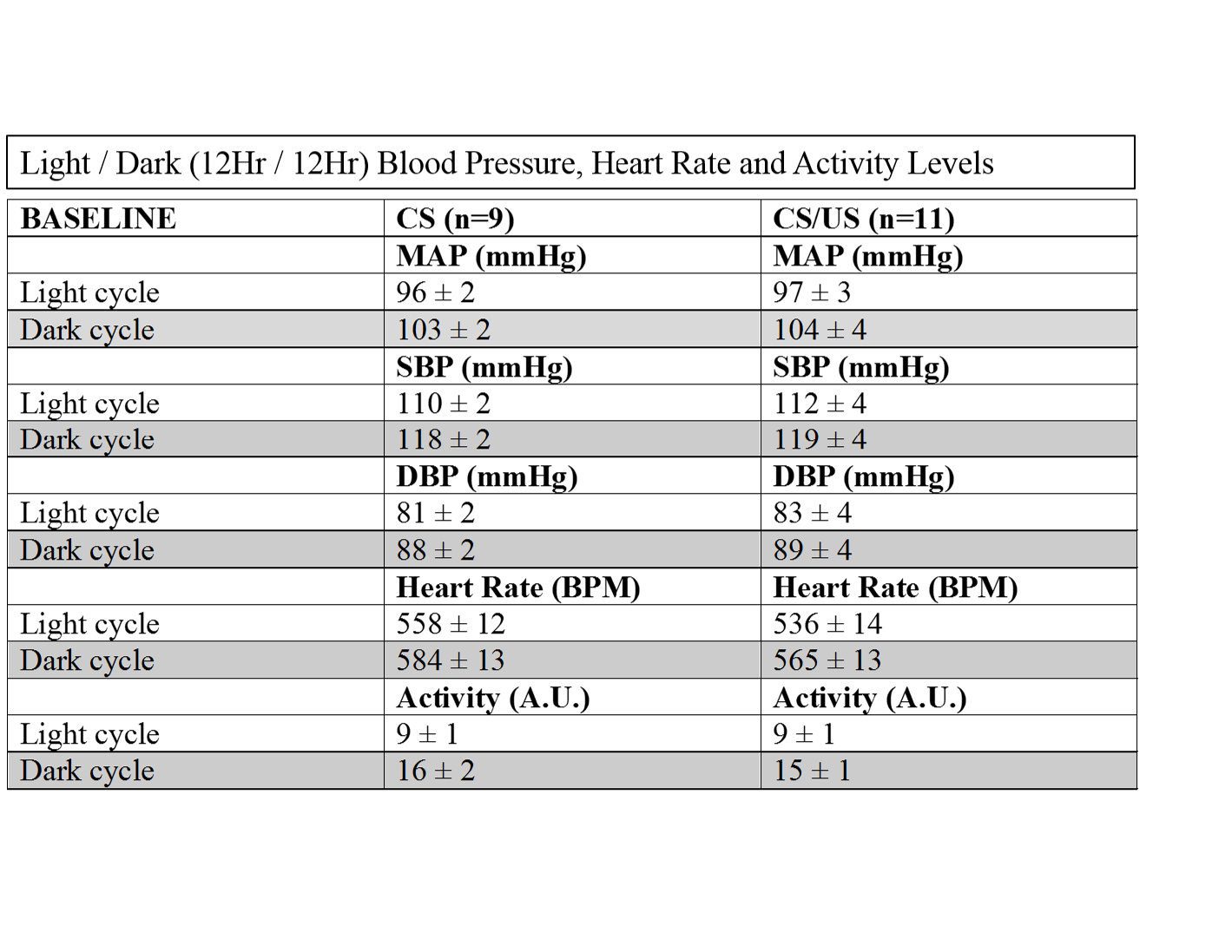

Supplement: TABLE S1 — Baseline day/night (12 h) mean arterial pressure (MAP), heart rate (HR), and activity levels in mice prior to fear conditioning. [file Image_3.TIF]
